# Driving Fatigue Onset and Visual Attention: An Electroencephalography-Driven Analysis of Ocular Behavior in a Driving Simulation Task

**DOI:** 10.3390/bs14111090

**Published:** 2024-11-13

**Authors:** Andrea Giorgi, Gianluca Borghini, Francesca Colaiuda, Stefano Menicocci, Vincenzo Ronca, Alessia Vozzi, Dario Rossi, Pietro Aricò, Rossella Capotorto, Simone Sportiello, Marco Petrelli, Carlo Polidori, Rodrigo Varga, Marteyn Van Gasteren, Fabio Babiloni, Gianluca Di Flumeri

**Affiliations:** 1 Department of Anatomical, Histological, Forensic and Orthopedic Sciences, Sapienza University of Rome, 00185 Roma, Italy; rossella.capotorto@uniroma1.it; 2 Department of Molecular Medicine, Faculty of Pharmacy and Medicine, Sapienza University of Rome, 00185 Roma, Italy; gianluca.borghini@uniroma1.it (G.B.); dario.rossi@uniroma1.it (D.R.); 3 Department of Physiology and Pharmacology, Faculty of Pharmacy and Medicine, Sapienza University of Rome, 00185 Roma, Italy; colaiuda.1783262@studenti.uniroma1.it (F.C.); stefano.menicocci@uniroma1.it (S.M.); fabio.babiloni@uniroma1.it (F.B.); 4 Department of Computer, Automatic and Management Engineering, Faculty of Information Engineering, Computer Science and Statistics, Sapienza University of Rome, 00185 Roma, Italy; vincenzo.ronca@uniroma1.it (V.R.); pietro.arico@uniroma1.it (P.A.); 5 BrainSigns srl, Via Tirso, 14, 00198 Rome, Italy; alessia.vozzi@brainsigns.com; 6 Department of Civil Engineering, Computer Science and Aeronautical Technologies, Roma Tre University, 00154 Roma, Italy; simone.sportiello@uniroma2.it (S.S.); marco.petrelli@uniroma3.it (M.P.); 7 Department of Enterprise Engineering, University of Rome Tor Vergata, 00133 Rome, Italy; 8 Italian Association of Road Safety Professionals (AIPSS), 00186 Rome, Italy; c.polidori@libero.it; 9 Instituto Tecnologico de Castilla y Leon, 09001 Burgos, Spain; rodrigo.varga@itcl.es (R.V.); marteyn.vangasteren@itcl.es (M.V.G.)

**Keywords:** EEG, eye tracking, fatigue, human factors, road safety, simulated driving, visual attention

## Abstract

Attentional deficits have tragic consequences on road safety. These deficits are not solely caused by distraction, since they can also arise from other mental impairments such as, most frequently, mental fatigue. Fatigue is among the most prevalent impairing conditions while driving, degrading drivers’ cognitive and physical abilities. This issue is particularly relevant for professional drivers, who spend most of their time behind the wheel. While scientific literature already documented the behavioral effects of driving fatigue, most studies have focused on drivers under sleep deprivation or anyhow at severe fatigue degrees, since it is difficult to recognize the onset of fatigue. The present study employed an EEG-driven approach to detect early signs of fatigue in professional drivers during a simulated task, with the aim of studying visual attention as fatigue begins to set in. Short-range and long-range professional drivers were recruited to take part in a 45-min-long simulated driving experiment. Questionnaires were used to validate the experimental protocol. A previously validated EEG index, the MDrow, was adopted as the benchmark measure for identifying the “fatigued” spans. Results of the eye-tracking analysis showed that, when fatigued, professional drivers tended to focus on non-informative portions of the driving environment. This paper presents evidence that an EEG-driven approach can be used to detect the onset of fatigue while driving and to study the related visual attention patterns. It was found that the onset of fatigue did not differentially impact drivers depending on their professional activity (short- vs. long-range delivery).

## 1. Introduction

Driving is one of the most common daily activities. A survey conducted in several European Union (EU) member states reported that 64% of daily trips are made by car [1]. The use of cars is even more frequent in the US, where an estimated 87% of the population drives daily [2]. It is not surprising that road injuries rank 12th among death causes globally [3], affecting people of all ages. When focusing specifically on the population between 5 and 29 years of age, road injuries score first among the causes of death. Already in 2018, the World Health Organization pointed out human factors as a crucial cause of 70% of car accidents, while it was recognized to be the main cause of accidents in 50% of the cases [4]. To respond to this, in the last decades, extensive efforts have been made to improve safety while driving [5,6]. In order to mitigate the number of accidents linked to the human factor, one possible solution consists of monitoring the driver’s psychophysical state in order to detect early symptoms of impairment. Fatigue and drowsiness are among the most studied impairing conditions, since they are very often experienced by people while driving. A survey published in 2018 highlighted how 30.8% of respondents drove at least once being too tired to keep their eyes open [7]. The impact of driving fatigue is so evident that both academic and industrial sectors are working together to establish validated methods to detect fatigue and drowsiness.

In recent years, car manufacturers have begun installing driver and driving monitoring systems in vehicles in order to detect fatigue and prompt alerts to prevent car accidents [8]. In the automotive industry, Volvo, Ford, Volkswagen, and others are offering cars with built-in monitoring devices [8]. The market also offers stand-alone monitoring systems that can be installed on any car [8]. The development of these devices was made possible thanks to the extensive effort that researchers are putting into studying fatigue and drowsiness correlates [9,10,11,12,13,14,15]. The research aims mainly at using several sources of user-related data to recognize fatigue. One source of information is represented by different kinds of neurophysiological signals such as electroencephalography (EEG) [16,17], heart rate [18,19], skin sweating [12], and eyeblink behavior [20,21], to recognize a change in the homeostatic state of drivers due to fatigue and drowsiness. An additional source of information is represented by the driver’s behavior in terms of driving style [22,23,24], which has been shown to be affected by fatigue. This effort in investigating how to recognize fatigue is aimed at preventing fatal and non-fatal accidents by alerting the drivers as soon as the system detects a risk [8].

Another way to prevent car accidents would be to design and build road infrastructures that can avoid or mitigate fatigue occurrence, such as the use of rumble strips at carriageway lines. It was largely demonstrated that road geometry could play a crucial role in fatigue occurrence [25,26,27]. In particular, road monotony can induce fatigue and a decrease in vigilance [25,26,27]. In this regard, the highway seems to be the type of road that most induces fatigue, due to its monotonicity and reduced visual stimulation. On the one hand, increasing the geometric complexity of a highway scenario would increase the performance in a driving task [27]. On the other hand, an excessively complex driving environment might overstimulate drivers, distracting them from the driving task. Therefore, monitoring only the drivers’ neurophysiological responses and the resulting driving style might be not sufficient to develop mitigation strategies and solutions to intervene in road infrastructure. Decision-makers must be also aware of the effects of fatigue on visual attention in order to design a driving environment able to nudge drivers toward safer behaviors.

Visual attention can be studied through eye-tracking technologies [28]. Eye tracker devices track ocular movements, thus providing measures of the driver’s gaze distribution over the field of view. These systems might be wearable devices (mainly in research applications) as well as directly installed on the car cockpit (industrial solution). They usually consist of an infrared camera to detect eye parameters such as the position of the pupil, in order to understand where the visual attention is directed. The pupil position is a function of its movements. When the person is actually looking at something in particular, the pupil is not moving, and this condition is called *fixation* [28,29]. It can last from milliseconds (usually 200–300 ms) to several seconds, it is possible to easily detect it by an eye tracker, and it is generally recognized as a measure of attention. The fast movement of the pupil between two fixations is called *saccade*, which lasts between 30 and 80 ms. These are not the only movements detectable with eye tracking, but they represent the most used in the fields of psychology, ergonomics, and cognitive science. In eye tracking, the field of view is usually divided into subregions called areas of interest (AoIs) that represent a specific portion of what is visible. These metrics are then computed for each AoI in order to understand the allocation of visual attention towards different stimuli.

Most of the studies about driving fatigue and drowsiness adopt extensive experimental protocols, usually lasting 90 min or more [18,30,31,32]. These protocols are aimed at maximizing the chance of inducing fatigue in the participants. To this regard, then the common approach is to consider the first minutes as representative of the ‘Low Fatigue’ condition, while the last minutes are assumed a priori to be representative of the ‘High Fatigue’ condition for everyone. However, it would be more and more valuable to be able to recognize the early symptoms of fatigue while driving, i.e., its onset, rather than its late stage, in order to prevent fatal and non-fatal road accidents. Indeed, this critical point would enable the design of strategies and systems to mitigate the risks related to fatigued driving.

The aim of the present study is to investigate visual attention in professional van and truck drivers experiencing fatigue onset in a 45-min-simulated monotonous driving task. As mentioned above, the protocol usually adopted to study fatigue involves participants driving for 90 min or more. In our case, participants were asked to drive for a total of 1 h in order to induce a moderate level of fatigue rather than its severe state. In addition, instead of assuming a priori the last minutes as representative of the ‘High fatigue’ condition, another point of novelty of the study consisted of employing a previously validated EEG index, the MDrow [15,33], as an objective benchmark to precisely detect and label, at an individual level, the periods of the highest fatigue. Considering the non-extensive duration of the driving task, the highest level of fatigue was interpreted here as the onset of the fatigued state. Visual attention in the fatigued period was compared to visual attention at the beginning of the driving task to understand eventual differences in the allocation of visual attention when participants were fatigued. In particular, the following research questions (RQs) have been addressed:

(1) Does the visual attention of professional drivers change in a fatigued (vs. non-fatigued) condition?
(2) Does the kind of professional activity (short-range vs. long-range delivery) affect the impact of fatigue on visual attention while driving?

## 2. Materials and Methods

### 2.1. Participants and Experimental Setup

Nineteen (19) professional drivers (18 males, years old M = 37.3, SD = 8.7), with normal or corrected-to-normal vision, were recruited to take part in the study. The experiments took place in Italy and Spain, where were recruited van drivers (9) and truck drivers (10), respectively. The experiment was conducted following the principles outlined in the Declaration of Helsinki of 1975, as revised in 2008, and it was approved by the Sapienza University of Rome and Roma Tre Ethical Committee (nr. 2022/02/22/CE, signed on 22 February 2022). Experiments took place in the afternoon to increase the chance of inducing fatigue during the driving task [34].

### 2.2. Experimental Protocol

**Figure 1:**
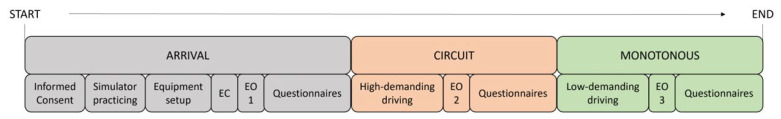
Description of the experimental protocol.

The research platform was a driving simulator constituted of a real car seat, a real dashboard with steering wheel, manual gearshift, and pedals and a 3-monitors-based display with a 160° view. The adopted experimental protocol is depicted in Figure 1. After an initial training phase, the neurophysiological activity in the resting state was collected. Participants were asked to sit and close their eyes for 1 min (‘Eyes Closed’ [EC] condition). Then they were instructed to look at the main monitor for 1 min without performing any task (‘Eyes Open’ [EO] condition, EO1). Subsequently, participants were provided for the first time with questionnaires for the self-assessment of their perceived fatigue (described below).

After this initial phase, participants had to drive in two simulated environments, a challenging and a monotonous one, in a fixed order, according to what was suggested by scientific literature [26,35]. Specifically, fatigue is supposed to be promoted by an intense task load, followed by a low stimulating and monotonous task [26,35]. The designed protocol constituted the first high-demand driving simulation whose aim was to challenge the mental resources of the participants. The second simulated environment was designed to be extremely easy and repetitive. The first simulated environment consisted of a 15 min high-demanding driving task on a racetrack (hereinafter named ‘Circuit’). After the ‘Circuit’ task, the ‘Eyes Open’ condition (EO2) was again performed, and participants were provided with the second round of questionnaires. After that, the ‘Monotonous’ driving task lasted 45 min and it consisted of driving in an easy and repetitive path, reproducing urban road infrastructure, without traffic. The speed limit was set at 40 km/h. At the end of the monotonous driving task, the last ‘Eyes Open’ condition (EO3) and questionnaire phase were performed. A scheme depicting the entire experimental protocol is given below.

**Figure 2:**
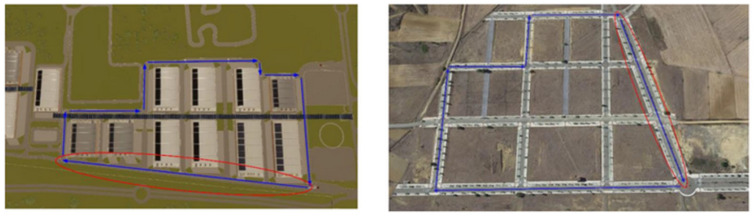
The two driving scenarios adopted in this study (**left**: van drivers, **right**: truck drivers). In order to reduce the noise in the data, statistical analysis was performed only on the data collected while participants were driving in the longest straight line (circled in red). Blue arrows indicate the direction while driving.

Experiments took place in two different locations, Spain and Italy, and they involved two different groups of participants. In Italy, van drivers were recruited, while in Spain, truck drivers were recruited. This approach was imposed by the objectives of the funding project (i.e., EU H2020 project ‘FitDrive’, GA n. 953432), in which it was planned to perform a follow-up of this study in which the same participants will be recruited to perform the same task but in real driving scenarios. For this reason, first, the real driving scenario locations were selected and then they were reproduced in the simulated environment. The maps of the two scenarios are depicted in Figure 2. As shown, the van location (left) and the truck location (right) consisted of a comparable number of turns and straight lines, resulting in a similar level of task difficulty. Since the two groups were different in terms of work experience and habits, i.e., van drivers are used to make short-distance deliveries, while truck drivers are used to driving for longer periods, it was possible to investigate whether this factor may have an impact on the obtained results (second research question).

### 2.3. Subjective Assessment

Two questionnaires were adopted to validate the experimental designs collecting perceived feelings of fatigue and sleepiness. Karolinska Sleepiness Scale (KKS) [36] and Chalder Fatigue Scale (Chalder) [9] were presented at the arrival and after each driving task for fatigue rating. From a conceptual and psychological point of view, mental fatigue and drowsiness are slightly different, even if usually they are considered just as two different degrees of intensity on a scale from alertness to sleepiness [37]. The choice to ask the participants to fill out both questionnaires was made because, being fatigue and sleepiness contiguous phenomena, they are often hard to distinguish between each other, especially if considering the poor sensitivity of subjective measures. In order to avoid participants misleading the symptoms as well as their interpretation, we provided a commonly used measure for fatigue in combination with a measure for sleepiness.

#### 2.3.1. Karolinska Sleepiness Scale

KSS [36] asks participants to rate their current state of sleepiness on a scale from 1 (extremely alert) to 9 (extremely sleepy—fighting sleep). This scale measures the subjective level of sleepiness at a particular time during the day, and therefore, it is sensitive to fluctuations.

#### 2.3.2. Chalder Fatigue Scale

Chalder questionnaire [9] asks participants to answer several questions about fatigue-related symptoms on a scale from 0 (none) to 3 (very high). In the original form, Chalder questions refer to two different dimensions called ‘physical symptoms’ and ‘mental symptoms’. Given the focus of this study (i.e., mental fatigue), only the questions related to this dimension were used (questions from 9 to 14 of the original questionnaire).

### 2.4. Electroencephalographic Assessment

EEG signal was collected using Mindtooth device (developed and validated during the EU H2020 Fast-Track-to-Innovation project Mindtooth, GA, USA) [38,39]. It consists of 8 Ag/AgCl electrodes with water-based sponges (so avoiding constraints imposed by the use of gel) placed according to the International 10-10 system [40] (AFz, AF3, AF4, AF7, AF8, Pz, P3, and P4) plus ground and reference electrodes placed on mastoids. The device has been validated and is capable of recording the EEG signal extremely accurately [39]. The sampling frequency was 125 Hz. To remove interferences due to mainline power interference a 50 Hz notch filter was applied. The EEG recordings were also band-pass filtered (low-pass filter cut-off frequency: 40 Hz, high-pass filter cut-off frequency: 2 Hz). Subsequently, Reblinca [41] algorithm was used to remove eyeblink artifacts while for other sources of artifacts, dedicated algorithms of the EEGLAB toolbox [42] were applied. The processed signal was then separated into 1 s-long epochs and the EEG epochs with the signal amplitude exceeding ±200 mV (threshold criterion) were labeled as ‘artifacts’ and removed from the EEG dataset with the aim of having a clean EEG signal to perform the analyses. We estimated an average of 21% (±22.51) of data loss due to the artifact rejection procedure.

The Global Field Power (GFP) was calculated from the artifact-free EEG with a focus on the EEG frequency band for the mental state of interest, which was the Alpha band [15]. The GFP was chosen because it describes brain EEG activity with the advantage of representing, in the time domain, the degree of synchronization of a specific cortical region of interest in a specific frequency band [43]. In fact, it is computed as the sample-by-sample averaged energy of the EEG signals coming from the electrodes representing the cortical area of interest, previously filtered into the selected frequency band. According to the Individual Alpha Frequency (IAF) value [44], the Alpha band was computed for each participant. Since the Alpha peak is mainly prominent during rest conditions, the subjects were asked to keep their eyes closed for a minute before starting the experiment. Such a condition was then used to estimate the IAF value specifically for each participant. Consequently, an EEG “strict” Alpha band was defined as Alpha = (IAF − 1):(IAF + 1) Hz. This definition of the Alpha band is more restrictive (thus “strict”) compared to the vast majority of Alpha band definitions that can be found in scientific literature, which is (IAF − 2):(IAF + 2) Hz. This approach was selected according to Klimesch [45], who demonstrated that a tighter band around the IAF can be considered as Alpha to avoid the impact from closer EEG frequency bands (Theta and Beta) variations on the observed phenomena in the Alpha band. The GFP was calculated over all the EEG parietal channels for each epoch using a Hanning window of the same length of the considered epoch (1 s length, which means 1 Hz of frequency resolution). The EEG was used to compute a Mental Drowsiness index (MDrow) [15], which is based on the increased Alpha GFP on parietal regions as a feature to detect fatigue. The MDrow was then used to (i) assess the level of mental fatigue immediately before and after each experimental task (EO conditions) and (ii) detect the time windows in which each participant experienced the highest level of fatigue during the Monotonous driving (see Section 2.6 for a detailed description of the analysis design).

### 2.5. Ocular Assessment

**Figure 3:**
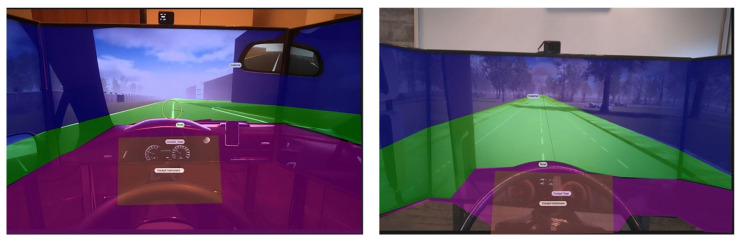
Representation of the AoIs designed for both van (**left**) and truck (**right**) drivers. Green: Road; Orange: Cockpit; Blue: External Environment; Purple: Cockpit Total (this is not discussed in this paper because of the neglectable amount of attention participants paid to this AoI).

Ocular behavior was measured by means of a research-grade wearable eye tracker, the Tobii Pro Glasses 2. Data were collected with a sampling frequency of 100 Hz through two infrared cameras mounted on the device. A calibration phase was performed before starting each data collection in order to check and adjust the quality of the data being collected. Epochs with low data quality due to poor light conditions, excessive motion, or any other impairing reason were automatically discarded from the Tobii Pro Lab software (v1.95.14258). The ocular assessment was based on the visual attention drivers paid to several areas of interest (AoIs), i.e., the different regions of their field of view. In general, the field of view of the user is divided into different AoIs that have common and coherent features and that can be considered as ‘points of interest’ for the user’s gaze. The identified AoIs are depicted in Figure 3, and each AoI was designed to capture a specific portion of the field of view while driving. In particular, the following AoIs were designed:

- Road: including the road infrastructure (Figure 3, in green);
- Cockpit: including the speedometer area (Figure 3, in orange);
- External Environment: including the external environment that is not the road (Figure 3, in blue);
- Cockpit Total: including the whole cockpit (Figure 3, in purple). For the analysis, this AoI was not considered because it contained information that was not related to the driving task.

The aim of this assessment was to investigate visual attention while driving under fatigue. To measure ocular behavior correlates of fatigue, several metrics were computed. These metrics are described in Table 1, and they are based on the concepts of fixation and visit. As mentioned in the introduction, fixation is the period in which the pupil is not moving, when participants are looking at something. The other metric considered, visit, is a metric computed by Tobii Pro Lab, and it is defined as the sum of all the continuative saccades and fixations within the same AoI.

### 2.6. Statistical Analysis

When analyzing questionnaires, we refer to the experimental conditions as ‘Arrival’ (data collected at participants’ arrival), ‘Circuit’ (data about the first driving task in the circuit), and ‘Monotonous’ (data about the second driving task in the monotonous environment). Questionnaires were analyzed with the non-parametric test. In particular, when comparing the three conditions, the Friedman test has been performed. In case of significant overall effect, pairwise comparisons between conditions have been performed by means of a post hoc test. The reported ‘p’ parameter of significance has been always corrected for multiple comparisons by the Bonferroni–Holm method. When analyzing eventual differences in van drivers compared to truck drivers, two separate *t*-tests were performed because the Friedman test with the grouping factor (VEHICLE, 2 levels (Van and Truck)) is not recommended. Then, the results of the two tests were compared in order to verify the similarity in the results. If the two groups showed the same significant comparisons, this was taken as proof of no difference between the two groups.

EEG data were used to compute the MDrow index to detect and label the time windows in which each participant experienced the highest level of fatigue (‘High fatigue’ condition), instead of assuming a priori that the last segment of the driving task was the period of highest fatigue everyone. A crucial aspect of this study is that the analysis was performed after the variability in the experimental data was reduced. The variability is represented by the fact that, considering the whole 45 min ‘Monotonous’ task, when detecting the highest level of fatigue, it could happen that participants were performing different activities (i.e., they could be either driving straight or turning left or right). For this reason, the position of the vehicle along the experimental driving path was used to isolate those moments in which participants were driving on the longest straight section of the path (circle in red in Figure 2). The EEG assessment was performed only on this homogeneous data in order to reduce the variability between participants at the minimum level. This is especially important when considering ocular behavior which is affected by the activity performed. After reducing data variability, the ‘High fatigue’ condition was compared with the first instance of participants driving on the longest straight line at the beginning of the driving task (‘Low fatigue’ condition). It was assumed that at the beginning of the driving task, participants experienced the lowest level of fatigue. Gaussian distribution of considered measures was verified using the Shapiro–Wilk Test. If normality was confirmed, a paired sample parametric test was performed, otherwise unpaired sample non-parametric test was used.

In order to further validate the adoption of the MDrow index, statistical analysis was performed on its scores measured over the resting state activity (ANOVA, factor RESTING STATE, 3 levels [EO1, EO2, and EO3]), assuming an index increasing from EO1 to EO3.

After this, to answer RQ1, ocular behavior (*t*-test, factor FATIGUE, 2 levels [‘Low fatigue’ and ‘High fatigue’]) was analyzed. In order to answer RQ2, the significance of the interaction of both factors FATIGUE and RESTING STATE with the factor VEHICLE was checked.

## 3. Results

### 3.1. Behavioral Assessment

#### 3.1.1. Karolinska Sleepiness Scale

**Figure 4:**
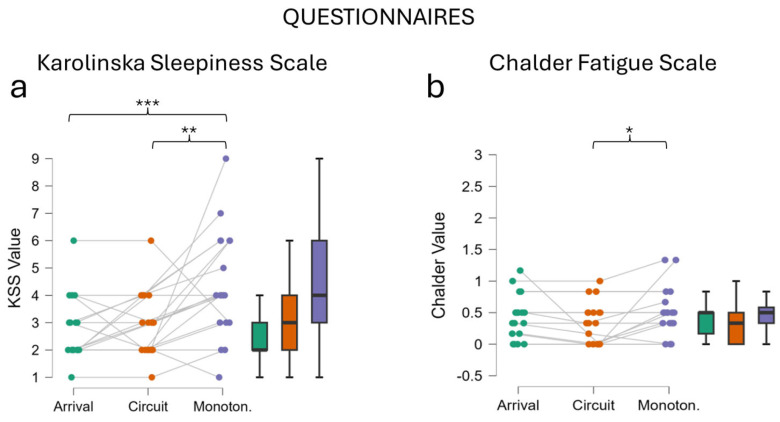
Results of questionnaires analysis. Participants perceived higher levels of both sleepiness (**a**) and fatigue (**b**). The choice of providing both questionnaires was based on the fact that fatigue and sleepiness might be difficult to distinguish between each other. * *p* < 0.05; ** = *p* < 0.01; *** *p* < 0.001.

KSS questionnaire analysis showed an overall significant effect, with the level of perceived sleepiness increasing across the experimental protocol (Friedman Test Chi-Squared = 16.871, df = 2, *p* < 0.001). As shown in Figure 4a, the post hoc tests showed that after the Monotonous driving task, participants reported higher level of sleepiness compared to both the moment of the Arrival and after the Circuit driving task (Conover Test ‘Arrival’ vs. ‘Monotonous’ T-Stat = 3.886, df = 36, *p* < 0.001; Conover Test ‘Circuit’ vs. ‘Monotonous’ T-Stat = 3.163, df = 36, *p* = 0.02). In particular, the drivers who experienced mental fatigue increased from an average score of 3 (before) to an average score of almost 5 after its conclusion. While a KSS score equal to 3 is considered ‘Alert’, a KSS score equal to 5 is considered ‘Neither alert nor sleepy’, while scores higher than 5 are related to the less or more effects of sleepiness, supporting the hypothesis of alertness decreasing as a prodrome of fatigue onset.

#### 3.1.2. Chalder Fatigue Scale

Chalder Fatigue Scale analysis revealed a significantly higher level of perceived mental fatigue (Friedman Test Chi-Squared = 7.806, df = 2, *p* = 0.02, Figure 4b). The post hoc analysis pointed out that the fatigue experienced after the Monotonous driving task was significantly higher with respect to the Circuit driving task (Conover Test T-Stat 2.914, df = 36, *p* = 0.02), while no significant differences arose from the comparison between Monotonous and Arrival (Conover Test T-Stat = 1.457, df = 36, *p* = 0.30).

### 3.2. EEG Assessment

**Figure 5:**
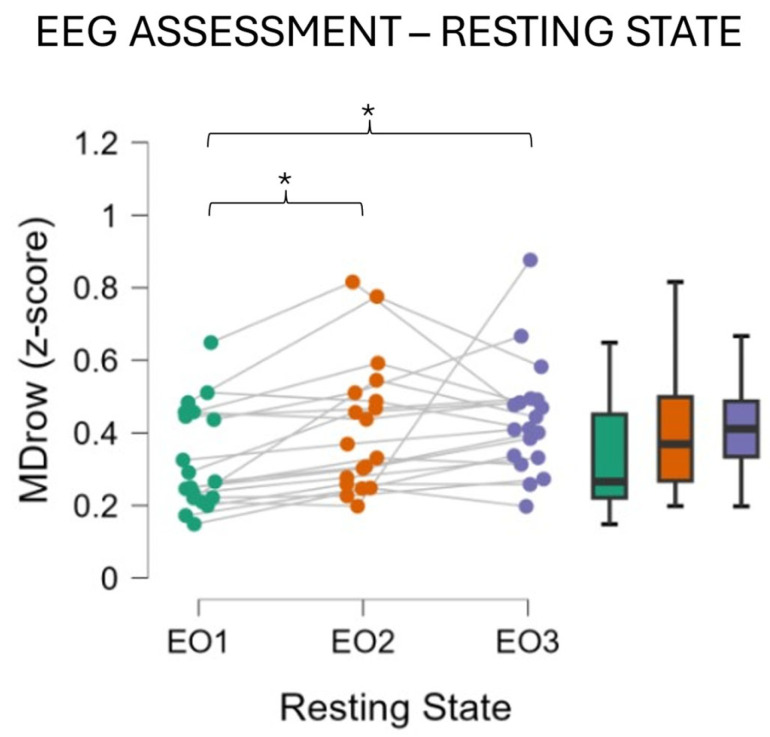
EEG assessment during the resting state collected at the participants’ arrival and after each driving task. As shown, after the circuit driving task (EO2), participants experienced an increase in fatigue that was found to be further higher after the monotonous driving task (EO3). * *p* < 0.05.

The analysis of the EEG-based MDrow index collected during ‘Eyes Open’ conditions just before and after each driving task, performed as a validation of the MDrow index sensitivity toward the mental fatigue increasing, revealed an increase in mental fatigue after the Circuit driving task (Figure 5), which remained high even after the Monotonous driving task (EO3).

### 3.3. Ocular Assessment

#### 3.3.1. Road AoI

Analysis of the driving-related AoI (i.e., Road) highlighted a tendency to a reduced number of both Fixations and Visits during the ‘High fatigue’ condition, but this reduction was not significant.

#### 3.3.2. Cockpit AoI

**Figure 6:**
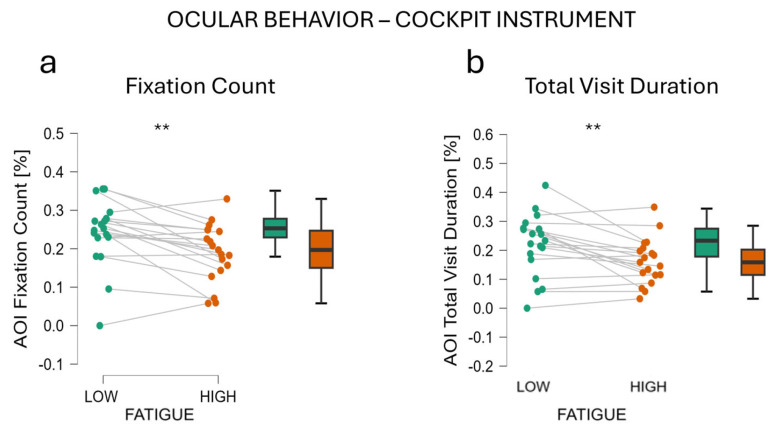
Analysis of ocular behavior during Low vs. ‘High fatigue’ condition. Subfigures (**a**,**b**) respectively show Fixation Count and Total Visit Duration. Both these measures decreased when participants were fatigued. ** represents *p* < 0.01.

Also, the analysis of the Cockpit AoI (Figure 6) highlighted a significant difference between ‘Low fatigue’ and ‘High fatigue’ conditions. When fatigued, participants showed a reduced number of Fixations (‘Low fatigue’ vs. ‘High fatigue’ *t*-test t = 3.515, df = 18, *p* = 0.002, Cohen’s d = 0.806, Figure 6a) to the instrumented portion of the cockpit (Low fatigue’ Fixation Count M = 0.190, SD = 0.075; ‘High fatigue’ Fixation Count M = 0.243, SD = 0.086).

Analysis revealed an impact of fatigue onset on the Total Visit Duration (‘Low fatigue’ vs. ‘High fatigue’ *t*-test t = 3.367, df = 18, *p* = 0.003, Cohen’s d = 0.773, Figure 6) to the instrumented portion of the cockpit (‘Low fatigue’ M = 0.220, SD = 0.106; ‘High fatigue’ M = 0.162, SD = 0.079).

#### 3.3.3. External Environment AoI

**Figure 7:**
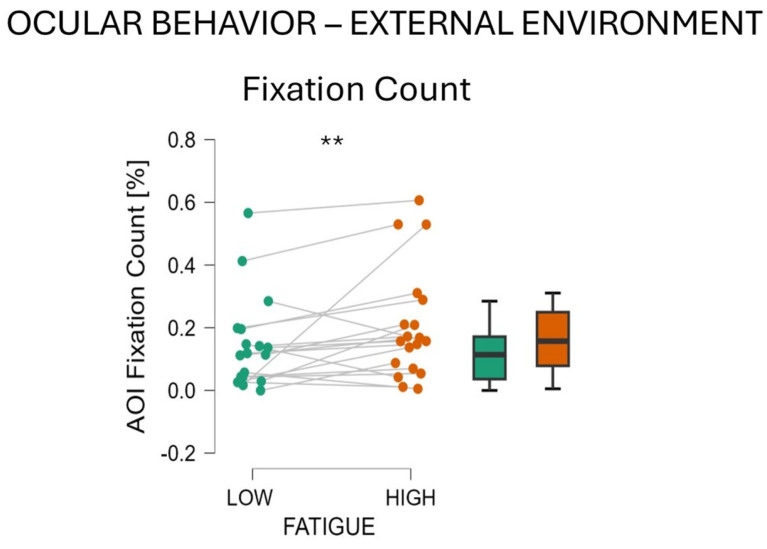
Analysis of ocular behavior toward External Environment during ‘Low fatigue’ vs. ‘High fatigue’ condition. Fixation Count has been found to decrease when participants were fatigued. ** represents *p* < 0.01.

During the ‘High fatigue’ condition, participants showed an increased number of Fixations in the External Environment AoI (‘Low fatigue’ vs. ‘High fatigue’ *t*-Test t = −2.106, df = 18, *p*= 0.05, Cohen’s d = −0.483, Figure 7) (‘Low fatigue’ M = 0.141, SD = 0.146; ‘High fatigue’ M = 0.205, SD = 0.177). Also, the Total Visits Duration was found to increase, but this increase was not significant.

### 3.4. Difference Between Van and Truck Drivers

As mentioned in Section 2.6, for questionnaires, two separate Friedman ANOVA were performed to study van drivers and truck drivers separately. In this case, we did not directly compare the response of van vs. truck drivers. We rather investigated if an eventual differential response was present; in other words, if the two groups showed different significant or not significant results. When analyzing MDrow during resting state conditions (EOs) and each of the ocular parameters, we checked the distribution of the samples, and the Gaussian distribution was confirmed. This allowed us to perform several two-way ANOVA analyses with two factors. The first was RESTING STATE × VEHICLE ANOVA, while the others were FATIGUE × VEHICLE ANOVA for each of the ocular parameters considered. Results obtained showed no effect due to an interaction with the factor VEHICLE.

## 4. Discussion

This paper presents the results of ocular behavior analysis on a group of professional drivers who performed a driving task aimed at inducing fatigue. The objective was to study visual attention when the onset of fatigue occurs, to better investigate one of the major causes of fatal and non-fatal accidents on the roads. Excluding substance-use-related accidents, it has been reported that fatigue, together with inadequate attention while driving, accounts for the majority of car crashes [46]. In this view, being able to understand how a fatigued mental state impacts visual attention while driving seems to be a crucial factor in preventing car accidents and increasing safety for both workers and citizens. A deeper knowledge of the effects of fatigue on driving would allow both the drivers to recognize fatigue and minimize its detrimental impact on driving ability and safety, and it would also help policymakers and car manufacturers in designing a better driving environment (i.e., roads, infrastructures, vehicles, on-board instrumentation) capable of nudging drivers toward safer behaviors, as well as new appropriate training procedures for fatigue management.

Three important aspects of the study are worth highlighting. The first aspect is related to the duration of the experiment and, therefore, to the resulting mental state that was investigated in this study. Most of the studies present in the literature on fatigued driving focused on prolonged driving tasks, resulting in the induction of a severe state of fatigue. The present study adopted a relatively short driving task (15 + 45 min) in order to induce a moderate level of fatigue (i.e., onset of fatigue). In fact, detecting and recognizing the early stages of fatigue appear to be the most effective way to intervene and improve safety while driving. Indeed, as most of the drivers monitoring systems do [8], being able to recognize a severe state of fatigue (i.e., drowsiness) does not prevent the possibility of car crashes because if the driver is showing heavy symptoms of fatigue, this means that it is already at risk. In this view, an optimal preventive strategy would consist of alerting the driver when their level of fatigue is about to be dangerous. The second important characteristic of this study is that the experiment was performed in highly realistic conditions, allowing a more reliable transposition of the findings to a real driving environment. To ensure high fidelity with a real driving condition, several aspects were considered: (a) professional drivers were recruited to take part in the study; (b) their age, as well as the gender proportion (1 female out of 19 participants), was representative of the European transport workers population [47]; (c) the simulated task was designed to reproduce a real area in the cities where experiments were performed, thus increasing the fidelity of the experimental setup; (d) experiments took place in the afternoon which, along with night, is when drivers, especially professional ones, experience the most acute and dangerous level of fatigue while driving. This time of the day was chosen to increase the chance of inducing fatigue. The last aspect that we would like to highlight is the process through which the variability in the data collected while driving was reduced to a minimum. The variability consisted of the fact that using the EEG as a benchmark to determine the highest level of fatigue means that participants might be actually performing a different activity in the detected time windows. This variability could be interpreted as noise interfering with the phenomenon being investigated in the analysis. To reduce the noise in the data, only EEG and ocular behavior collected, when participants were driving on the longest straight line of the Monotonous driving task, were considered. In this way, we were sure that all participants were performing the same activity—straight driving—reducing the bias in the data.

The results obtained showed that the experimental protocol successfully induced a fatigued mental state. Using the subjective reports collected with the KSS and Chalder questionnaires, we demonstrated that participants perceived an early fatigued mental state, especially after the Monotonous driving task (Figure 4). The choice of providing both questionnaires was motivated by the fact that fatigue and drowsiness, being contiguous and related phenomena, might be difficult to discriminate between each other. This choice was also supported by the duration of the experimental protocol, which was 1 h in total. It was assumed that in this time span, a severe state of fatigue (i.e., drowsiness) would unlikely be induced and thus, the impaired mental state eventually perceived would be attributed to fatigue rather than drowsiness. Consequently, the increase in both perceived fatigue and drowsiness was interpreted as a validation of the experimental design. The EEG assessment also supported the experimental design. This assessment was performed in order to further validate the feature used to compute the MDrow index, that is, the Alpha EEG band on the parietal region. This index was already validated in a previous study [15], but a check on its sensitivity along the adopted protocol was performed in order to ensure the reliability and consistency of the data presented in this paper. The EEG analysis of data collected during the resting states (Eyes Open conditions, Figure 1) demonstrated cortical correlates of fatigue in the form of increased Alpha activity on the parietal area after the Monotonous driving task.

The validation of the experimental design both with questionnaires and EEG assessment supported the adoption of the EEG index as a benchmark to detect the moments in which each participant experienced the highest level of fatigue (considered here as the onset of fatigue itself). The time windows detected with the MDrow index were then used to compute the eye tracker metrics for each of the designed areas of interest (AoIs). The ocular behavior data related to this ‘High fatigue’ condition were compared to the one collected the first time participants drove in the longest straight line of the Monotonous driving task. To study ocular behavior, three AoIs were considered: Road, Cockpit, and External Environment. These three AoIs were classified as providing a decreasing amount of information related to the driving task. In particular, the Road AoI (Figure 3, in green) provided the main source of information when driving, those related to the position of the car with respect to the street. Then, there was the Cockpit AoI (Figure 3, in orange), where participants could monitor the driving parameters related to the task, basically speed and engine status. This information was not vital for driving; but since the task also included driving at 40 km/h and responding to a secondary task mimicking an engine failure, participants were actually asked to pay attention to this portion of their field of view. The last AoI was the External Environment (Figure 3, in blue), which was defined as everything that is not included in the aforementioned AoIs. This AoI did not contain any relevant information for the drivers. We initially designed an additional AoI, where there should be some internal components of the vehicle, such as radio, controls, fans, and other accessories (Figure 3, in purple). Considering the negligible amount of attention that all participants paid to this AoI, we decided to not discuss it further.

Looking globally at the results, it emerged that when fatigued, participants shifted their visual attention from task-related information toward non-task-related information. Considering the main source of information related to the driving task, the Road, a tendency to a reduction in visual attention toward this AoI, was observed. Indeed, even if not significant, participants showed a reduced number of fixations and visits in this AoI. The reduction in visual attention was even more pronounced on the AoI that was providing useful, but not vital, information for the drivers. Indeed, a significant decrease was observed in both the number of fixations and the total duration of each visit in the Cockpit AoI during the ‘High fatigue’ condition compared to the ‘Low fatigue’ condition. This means that participants spend significantly less time monitoring their speed and the engine status when fatigued. As we mentioned, this information was not vital for driving the car but was useful to complete the task at its best. Participants were indeed asked to check and adjust their speed and to monitor the status of the engine in case any problem occurred. The same is true in real driving, where it is a good practice to monitor and check the status of the vehicle when driving even if monitoring is not the main task, which is driving. If we think of this monitoring activity as a secondary task and the actual driving of the vehicle as the primary task, we can interpret the data as how, when fatigued, drivers stopped attending the secondary task while keeping attending the primary one. This is in line with the literature, where it is largely demonstrated that when mental resources decrease the performance in a secondary task decreases accordingly [48,49,50]. Similar findings were highlighted by several studies on aircraft pilots [10,51]. Among these, Naeeri et al. [51] found that when fatigued, pilots showed a decreased number of fixations on the cockpit of the aircraft, indicating less cognitive processing in evaluating task-related information. At the same time, they found that fixation duration increased, attributing this to the impairment due to fatigue when processing the complexity of the aircraft cockpits. In our case, we confirmed the decrease in the fixation number on the cockpit (less information processing) when fatigue occurs. In contrast to what was found by Naeeri et al., we found a decrease in visits’ duration (consecutive fixations on the same AoI) during the ‘High fatigue’ condition. To account for this, it can be argued that the complexity of the simulated car cockpit in our experiment was very low (Figure 3), and for this reason, it did not request additional processing time, even when impaired by fatigue. A similar behavior was reported by Wu et al. [10]. These authors confirmed that during a simulated flight experiment, fatigue induced by the task was reflected in lower visual attention on the AoIs that were providing relevant information to complete the task; indeed, when fatigued participants showed a lower number of fixations to the same AoIs.

Lastly, we were interested also in investigating eventual differences in the considered measures depending on the different vehicles driven as well as the usual driving experience, hypothesizing that truck drivers could have been more resilient to mental fatigue. This hypothesis comes from the fact that truck drivers, compared to van drivers, are used to longer travel ranges during their work. This exposes them to a higher chance of experiencing fatigue, which in turn might lead to the development of regulatory responses. To do this, both van drivers and truck drivers were recruited to take part in the study. It must be noted that even if the two groups drove on two different experimental roads (Figure 2, van left, trucks right), data variability reduction has been performed, considering only the longest straight line of the roads (Figure 2, circled in red). This process ensured that when comparing the ocular behavior collected in different moments using the MDrow index (i.e., each participant’s ‘High fatigue’ condition), the eventual differences would be attributed only to fatigue occurrence rather than the fact that participants were performing different activities. The analysis revealed that no difference can be found between van and truck drivers for all the considered measures. This finding suggests that the effects of fatigue on visual attention can be generalized, and they do not depend on the type of vehicle being driven.

A limitation of this study was represented by the fact that experiments were conducted always in the afternoon. Although this was a deliberate choice, this might imply that during a different daytime (i.e., at night), the allocation of visual attention could change. Also, even if the experimental sample was representative of the European drivers’ population where nearly all the drivers are males, the gender proportion in this study was not balanced, not allowing a gender analysis to investigate differences between males and females. Future work should investigate these critical points. Also, an implementation of the present work should perform an entropy analysis, which was not included here. Another interesting aspect to investigate is the eventual difference between simulated and real driving, in order to extend the validity of the presented results outside of the laboratory environment.

## 5. Conclusions

This paper reported the visual attention changes related to the onset of fatigue occurring while driving. We performed an objective assessment of fatigue using a previously validated EEG mental fatigue index that was used as a benchmark to analyze the related ocular behavior. We have found that the onset of fatigue induced an allocation of visual attention toward non-informative areas of the field of view. At the same time, we found that the visual attention toward the main source of information, the road, remained statistically unchanged even if a tendency to decrease was visible. These results might point out directions in designing roads and driving infrastructures. We also demonstrated that the fatigue-induced changes in the allocation of visual attention while driving were not affected by the type of vehicle (van vs. truck) supporting the generalization of the findings.

## Figures and Tables

**Table 1:** Description of the metrics computed to measure visual attention on each AoI.

Metrics	Definition
Fixation Count	Number of fixations on the AoI
Average Fixation Duration	Average fixation duration on the AoI
Total Fixation Duration	Sum of all fixation duration on the AoI
Visit Count	Number of visits on the AoI
Average Visits Duration	Average visit duration on the AoI
Total Visits Duration	Sum of all visit duration on the AoI

## Data Availability

The data presented in this study are available on request at the moment due to restrictions. It will be made publicly available after the completion of the funding project.
